# Long non-coding RNA Malat1 promotes gallbladder cancer development by acting as a molecular sponge to regulate miR-206

**DOI:** 10.18632/oncotarget.9347

**Published:** 2016-05-13

**Authors:** Shou-Hua Wang, Wen-Jie Zhang, Xiao-Cai Wu, Ming-Di Zhang, Ming-Zhe Weng, Di Zhou, Jian-Dong Wang, Zhi-Wei Quan

**Affiliations:** ^1^ Department of General Surgery, Xinhua Hospital, Shanghai Jiao Tong University School of Medicine, Shanghai, 200000, China

**Keywords:** lncRNA, Malat1, miR-206, competing endogenous RNA, gallbladder cancer

## Abstract

Long non-coding RNA (lncRNA) metastasis-associated lung adenocarcinoma transcript 1 (Malat1) functions as an oncogene in many types of human cancer. In this study, we show that Malat1 is overexpressed in gallbladder cancer (GBC) tissue and cells. The high Malat1 levels correlated positively with tumor size and lymphatic metastasis, and correlated negatively with overall survival. We also show that Malat1 functions as a competing endogenous RNA (ceRNA) for miR-206. Because miR-206 directly suppresses expression of ANXA2 and KRAS, which are thought to promote GBC progression, Malat1 binding of miR-206 in GBC tissue and cells has an oncogenic effect. Conversely, Malat1 knockdown inhibits proliferation and invasion by GBC cells while increasing apoptosis. *In vivo*, silencing Malat1 decreases tumor volume. These results suggest Malat1 could potentially serve as a therapeutic target and prognostic marker for GBC.

## INTRODUCTION

Gallbladder cancer (GBC) is the fifth most common cancer involving the gastrointestinal tract and the most common malignant tumor of the biliary tract worldwide. Complete surgical resection is the only effective treatment; however, only 10% of patients are considered surgical candidates and recurrence rates after radical resection are high [2]. Indeed, the overall 5-year survival rate for GBC is less than 5% [1].

Metastasis associated lung adenocarcinoma transcript1 (Malat1) is a long non-coding RNA (lncRNA, > 200 nucleotides) first reported in non-small cell lung cancer (NSCLC) [3]. Malat1 may act as an oncogene in various types of cancer [4, 5, 6] and has been reported as a competing endogenous RNA (ceRNA) that regulates ZEB2 expression by sponging miR-200s in clear cell kidney carcinoma [7]. On the other hand, microRNAs (miRNAs) are small, non-protein coding transcripts (18– 25 nucleotides in length) that regulate mRNA translation [9]. MicroRNA-206 (miR-206) belongs to the group of so-called “myomiRs” [10] and is downregulated [11, 12, 13, 14] or upregulated [15, 16, 17] in various types of cancer.

Downregulation of Malat1 inhibits the proliferation and metastasis of GBC cells and inactivates the ERK/MAPK pathway [8]. Here we show that Malat1 is overexpressed in GBC tissue and functions as a ceRNA for miR-206. The sponging of miR-206 by Malat1 overexpression has oncogenic effects since miR-206 is no longer able to suppress downstream targets ANXA2 and KRAS, which are involved in GBC progression.

## RESULTS

### Malat1 levels are elevated in GBC tissues

**Figure 1:**
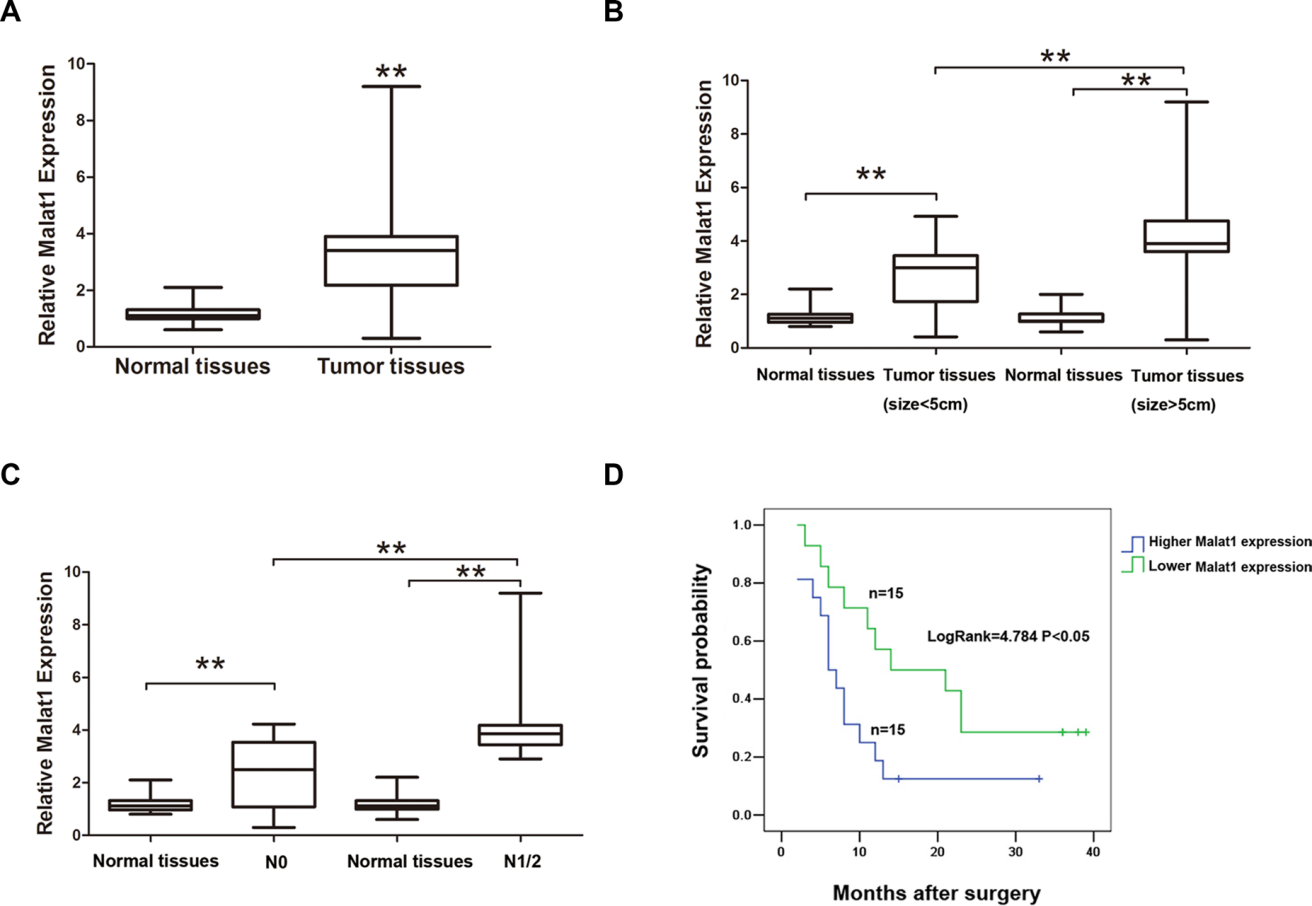
Malat1 expression and its clinical significance in GBC (**A**) Malat1 expression in pair samples of GBC and adjacent normal tissues (**P < 0.05). (**B**) Malat1 expression correlates positively with tumor size (**P < 0.05) and (**C**) lymphatic metastasis (**P < 0.05), while correlating negatively with (**D**) over survival (OS) time (Log Rank = 4.784, **P < 0.05).

**Figure 2:**
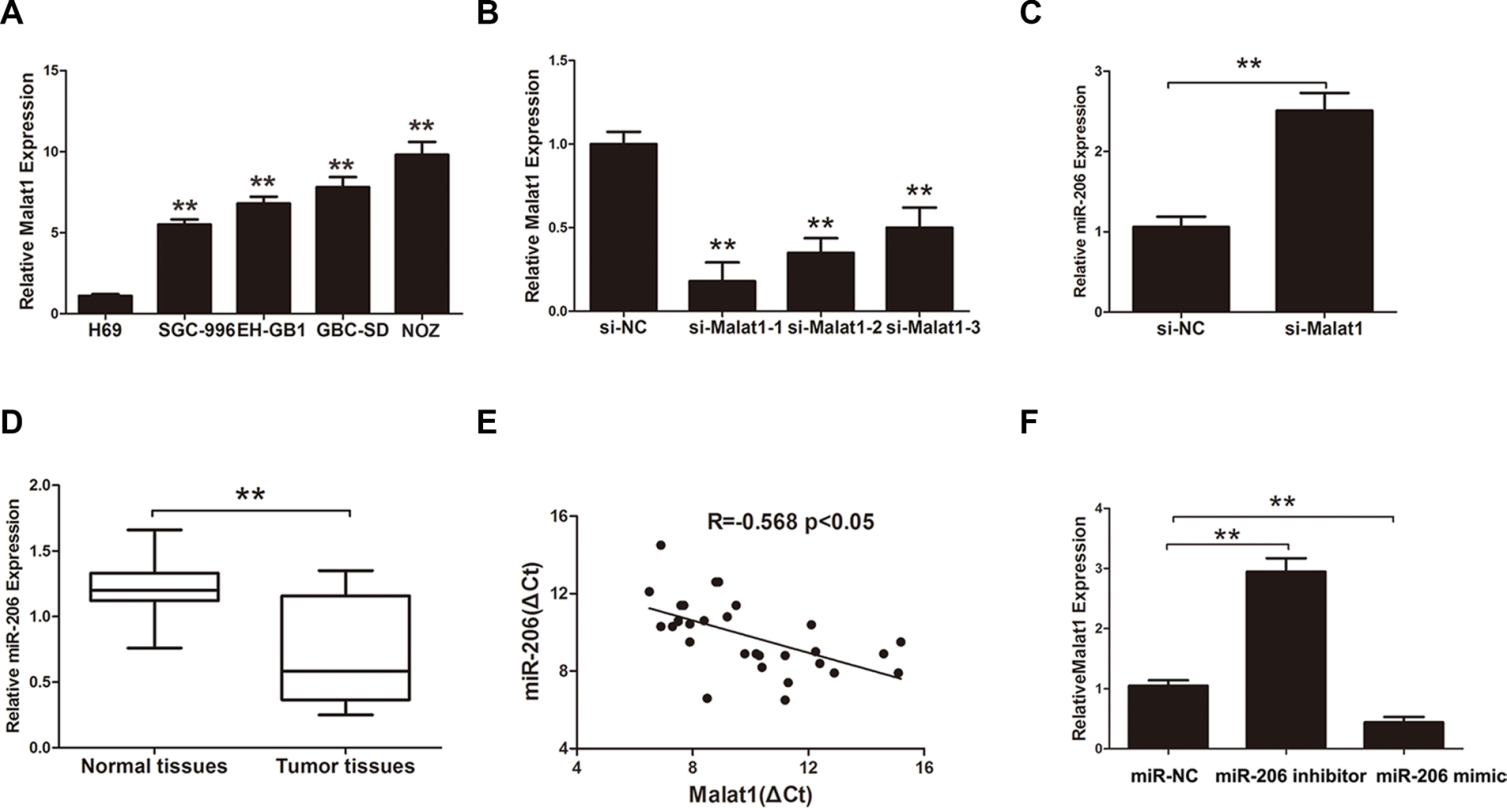
The correction between Malat1 and miR-206 in GBC (**A**) Relative levels of Malat1 in normal H69 cells and four types of GBC cells (**P < 0.05). (**B**) Efficacy of Malat1 interference in NOZ cells, (**P < 0.05). (**C**) MiR-206 expression after Malat1 knockdown in NOZ cells (**P < 0.05). (**D**) MiR-206 expression in GBC tissues (**P < 0.05). (**E**) Pearson's correlation between Malat1 and miR-206. (**F**) Malat1 expression after transfecting miR-206 mimic or miR-206 inhibitor. All data are represented as the mean ± S.D. from three independent experiments (**P < 0.05).

Malat1 levels were higher in GBC tissues than in normal gallbladder tissues (Figure 1A). Thirty GBC patients were classified into two groups depending on their Malat1 levels in tumor tissues relative to the median ratio (3.31): high-Malat1 group (*n* = 15, Malat1 expression ratio ≥ median ratio); and low-Malat1 group (*n* = 15, Malat1 expression ratio < median ratio). Upregulation of Malat1 correlated positively with tumor size (*P* = 0.013) and lymphatic metastasis (*P* = 0.005) while correlating negatively with overall survival (OS) (Figure 1B, 1C and 1D). No correlation was found between Malat1 upregulation and gender, age, histological grade or clinical stage (*p* > 0.05) (Table 1). In addition, the expression of Malat1 was upregulated in multiple GBC cell lines compared to normal biliary epithelial H69 cells (Figure 2A). On the contrary, relative miR-206 levels were lower in the same GBC cell lines than in H69 cells (Supplementary Figure S1).

**Table 1:** Correlation between Malat1 expression and clinical-pathological characteristic in 30 cases GBC patients

Clinical characteristics	Case number	Malat1 expression		*p*-value
		Low	High	
**Gender**				0.650
Male	10	5	5	
Female	20	10	10	
**Age**				0.450
≤ 60	19	11	8	
> 60	11	4	7	
**tumor size**				0.013^**^
< 5 cm	13	10	3	
> 5 cm	17	5	12	
**Histological grade**				
well and morderately	14	9	5	0.136
Poorly and others	16	6	10	
**N status**				
N0	14	11	3	0.005^**^
N1/2	16	4	12	
**Clinical stage**				0.132
I–II	12	8	4	
III–IV	18	7	11	

** *P* < 0.05.

### Identification of miRNAs that bind to Malat1

Previous studies have shown that lncRNAs function as ceRNA or “molecular sponges” to modulate miRNAs [18]. We used starbase 2.0 (http://starbase.sysu.edu.cn/) to find miRNAs that potentially bind to Malat1. Thirty-three predicted miRNAs were found by qRT-PCR after knockdown of Malat1 in NOZ cells (Supplementary Table S1). The interfering efficiency of Malat1 was measured by qRT-PCR (Figure 2B). MiR-206, a tumor suppressor gene in various types cancer [19], was chosen for further studies given its high upregulation (fold change = 2.78) in response to Malat1 knockdown in NOZ cells compared to the scrambled control (Figure 2C). Furthermore, miR-206 was downregulated in GBC tissues compared to match normal tissues (Figure 2D). Interestingly, Malat1 expression correlated negatively with miR-206 levels (*r* = −0.568, *P* < 0.05) (Figure 2E). Moreover, miR-206 inhibitor increased Malat1 levels in NOZ cells while miR-206 mimic decreased Malat1 expression (Figure 2F).

### Malat1 directly binds to miR-206

**Figure 3:**
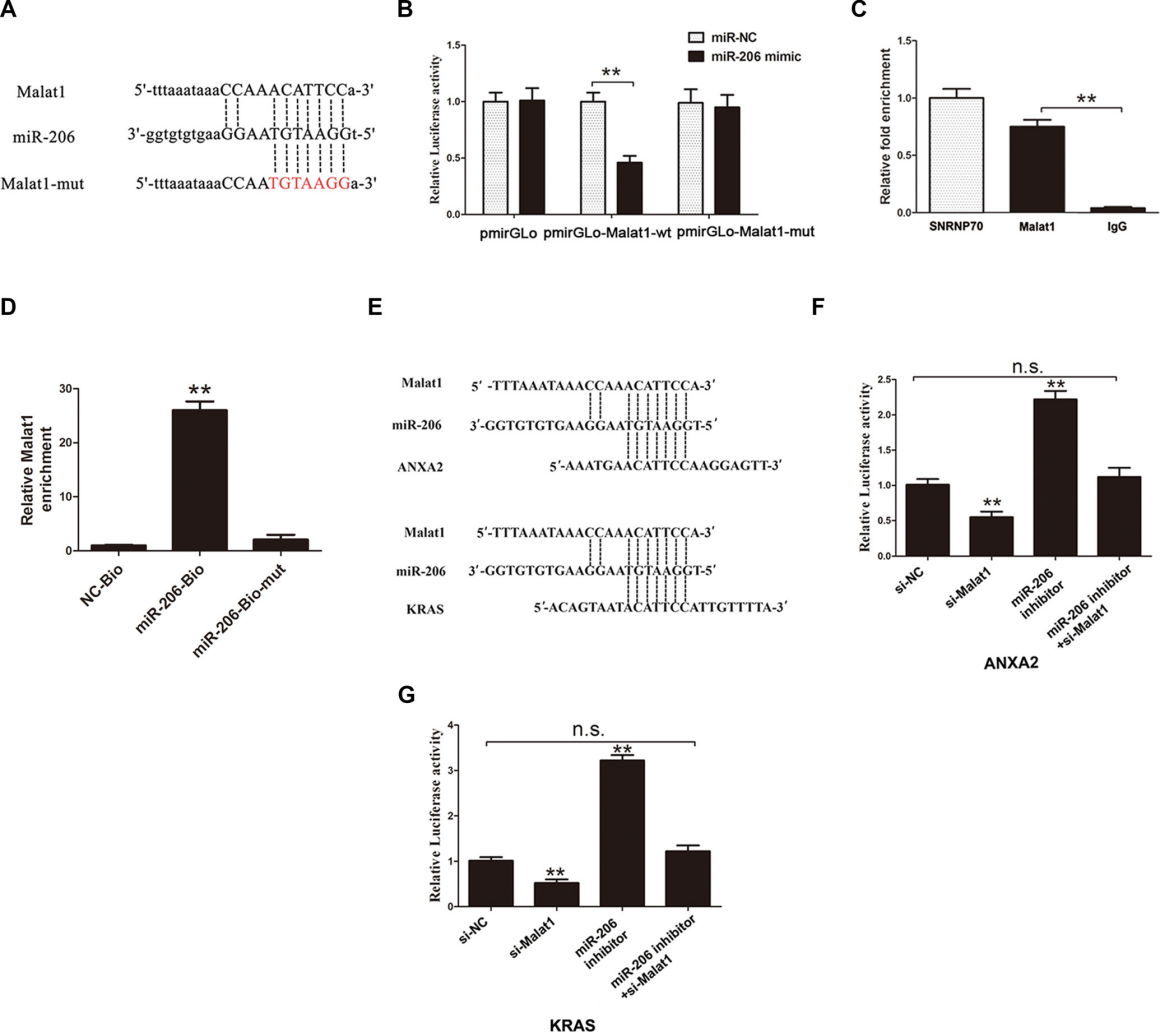
Direct interaction between Malat1/miR-206/KRAS or ANXA2 (**A**) MiR-206 binding sites on Malat1 predicted by Starbase v2.0. (**B**) Luciferase activity of the indicated group in HEK293T cells, (**P < 0.05). (**C**) Amount of Malat1 bound to Ago2 or IgG measured by RT–qPCR after RIP (**P < 0.05). (**D**) NOZ cells transfected with biotinylated WT miR-206 (miR-206-Bio) or biotinylated mutant miR-206 (miR-206-Mut-Bio) or biotinylated NC (NC-Bio), assayed by biotin-based pulldown 48 h after transfection. Malat1 levels were analyzed by RT–qPCR (**P < 0.05). (**E**) MiR-206 binding sites on ANXA2 and KRAS predicted by Starbase v2.0. (**F–G**) Luciferase activity of indicated groups in HEK293T cells (**P < 0.05).

We used dual-luciferase reporter assay to further investigate the interaction of miR-206 with Malat1. The fragment including the binding site as predicted by starbase (Figure 3A) was cloned into a pmirGLO vector as the wild-type (pmirGLO-Malat1-wt), so was the mutated fragment (constructed by replacing the binding site with its complementary sequence) (pmirGLO-Malat1-mut). MiR-206 mimic reduced the luciferase activity of pmirGLO-Malat1-wt but not of pmirGLO-Malat1-mut (Figure 3B). MiRNAs function through RNA induced silencing complex (RISC) [20]. Ago2 is an essential catalytic component of RISC involved in RNA cleavage [21]. RNA immunoprecipitation (RIP) indicated that Malat1 was preferentially enriched in Ago2-containing beads compared to those harboring control immunoglobulin G (IgG) antibody (Figure 3C, *P* < 0.05). U1 small nuclear ribonucleoprotein (SNRNP70, ~70 kDa), a gene coding SNRNP70 protein associated with U1 spliceosomal RNA [22], was used as a positive control. In addition, we performed pull down experiments using biotin-labeled miR-206 oligos. Malat1 was pulled down by biotin-labeled miR-206 oligos, but not the mutated oligos (binding sites were mutated to the complement sequences). (Figure 3D, *P* < 0.05). These results suggest that Malat1 directly binds to miR-206.

### Malat1 inhibits expression of endogenous miR-206 and increases levels of ANXA2 and KRAS

**Figure 4:**
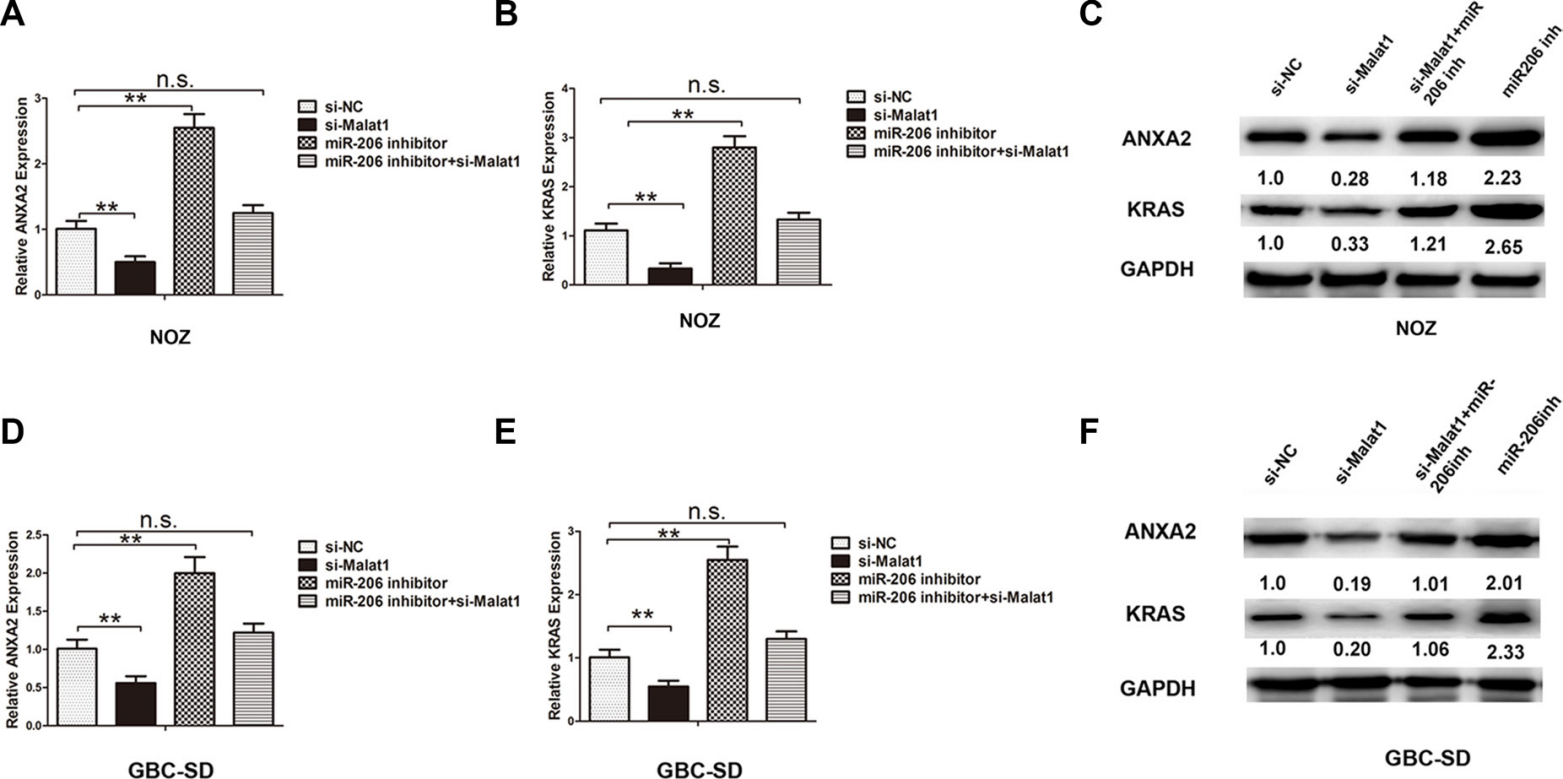
Knockdown of Malat1 inhibits expression of miR-206 targeting ANXA2 and KRAS The mRNA level of (**A**) ANXA2 and (**B**) KRAS, and protein levels of (**C**) ANXA2 and KRAS in four groups of NOZ cells: si-NC, si-Malat1, miR-206 inhibitor, and miR-206 inhibitor+si-Malat1. The mRNA level of (**D**) ANXA2 and (**E**) KRAS, and protein levels of (**F**) ANXA2 and KRAS in four groups of GBC-SD cells: si-NC, si-Malat1, miR-206 inhibitor, and miR-206 inhibitor+si-Malat1. All data are represented as the mean ± S.D. from three independent experiments (**P < 0.05).

MiR-206 has been reported as a tumor suppressor gene in human pancreatic ductal adenocarcinoma (PDAC), directly targeting oncogenes KRAS and annexin a2 (ANXA2) [23]. The transcriptional levels of ANXA2 and KRAS were both upregulated in GBC tissues compared to matched adjacent normal tissues (Supplementary Figure S2A, S2B). It has been hypothesized that ANXA2 and KRAS might promote turmorigenesis in GBC and play a role downstream of Malat1 and miR-206. To test whether Malat1 acts as a sponge of miR-206 and promotes ANXA2 and KRAS expression, luciferase reporter plasmids containing the 3′-UTR of ANXA2 and KRAS were constructed (Figure 3E). Malat1 knockdown decreased luciferase activity in HEK293T cells transfected with Luc-ANXA2-3′-UTR and Luc-KRAS-3′-UTR respectively. On the other hand, luciferase activity was rescued by miRNA-206 inhibitor in both cases (Figure 3F, 3G). Moreover, knockdown of Malat1 decreased transcriptional levels of ANXA2 in NOZ (Figure 4A) and GBD-SD cells (Figure 4D), while inhibiting ANXA2 protein expression (Figure 4C, 4F). However, this inhibition was attenuated by co-transfection of miR-206 inhibitor (Figure 4A, 4C, 4D, 4F). Malat1 knockdown also inhibited KRAS (Figure 4B, 4C, 4E, 4F). These results indicate that Malat1 might increase ANXA2 and KRAS by inhibiting miR-206 in GBC.

### Malat1, ANXA2 or KRAS knockdown inhibits proliferation and increases apoptosis in NOZ cells

**Figure 5:**
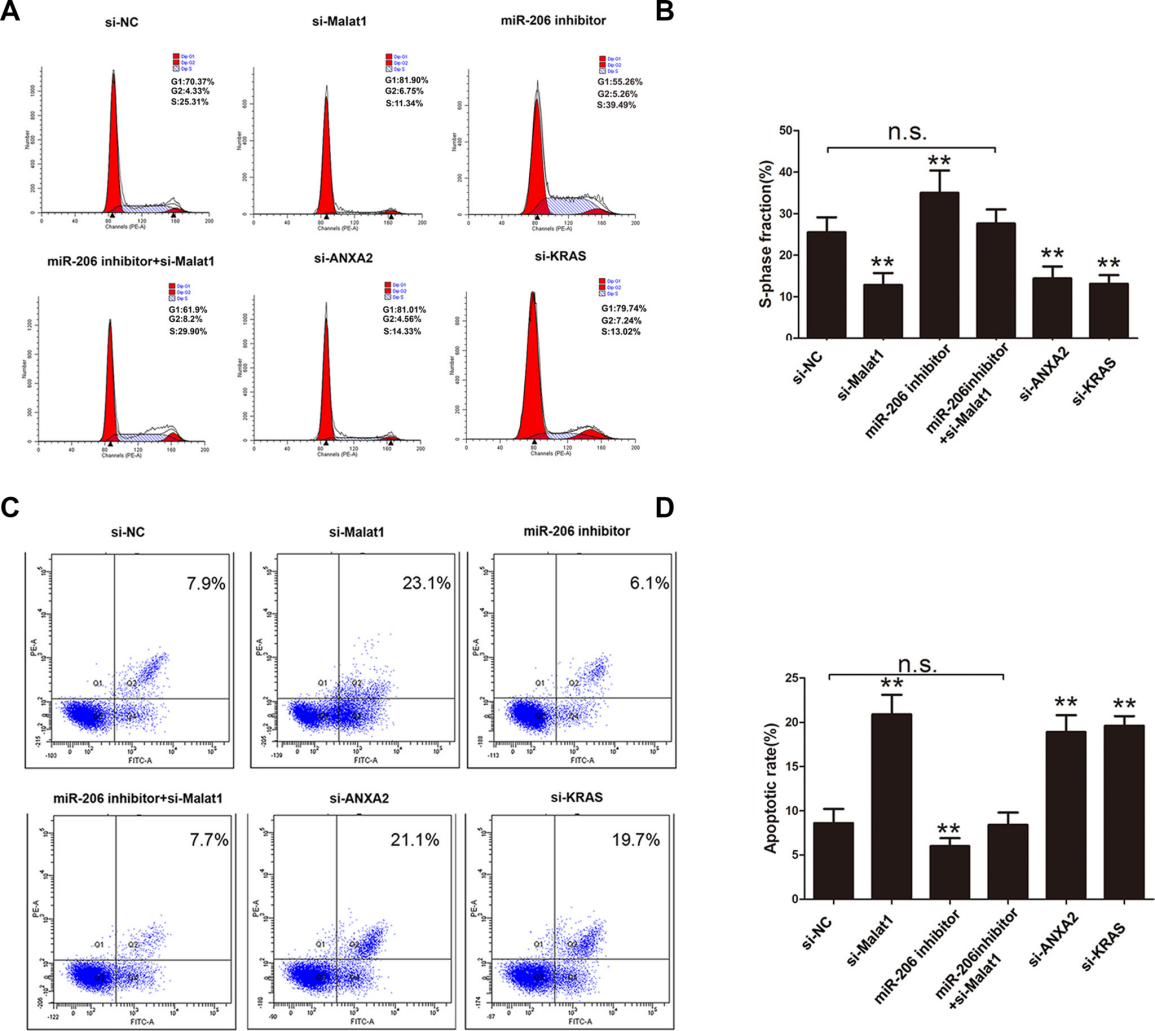
Cell cycle and apoptosis measured after silencing Malat1/KRAS or ANXA2 signaling (**A**) Cell cycle phase determination and (**B**) corresponding statistical summary, (**C**) cell apoptosis and (**D**) corresponding statistical summary, in NOZ cells transfected with si-NC, si-Malat1, miR-206 inhibitor, miR-206 inhibitor+si-Malat1, si-ANXA2 and si-KRAS. All data were represented as the mean ± S.D. from three independent experiments, **P < 0.05.

To determine the role of Malat1 in GBC development, the Malat1 gene was silenced in NOZ cells to assess its effect on cell proliferation, apoptosis and invasion. The percentage of NOZ cells in S-phase decreased after Malat1 siRNA transfection, while the reduction of expression was rescued by co-transfection of miRNA-206 inhibitor. Moreover, knockdown of ANXA2 or KRAS also reduced the percentage cells in S-phase (Figure 5A, 5B). Cell apoptosis was elevated after Malat1 knockdown as measured by anexinV-PI assay, and rescued by miR-206 inhibitor. ANXA2 or KRAS knockdown also increased cell apoptosis (Figure 5C, 5D).

### Malat1, ANXA2 or KRAS knockdown inhibits NOZ cell invasion

**Figure 6:**
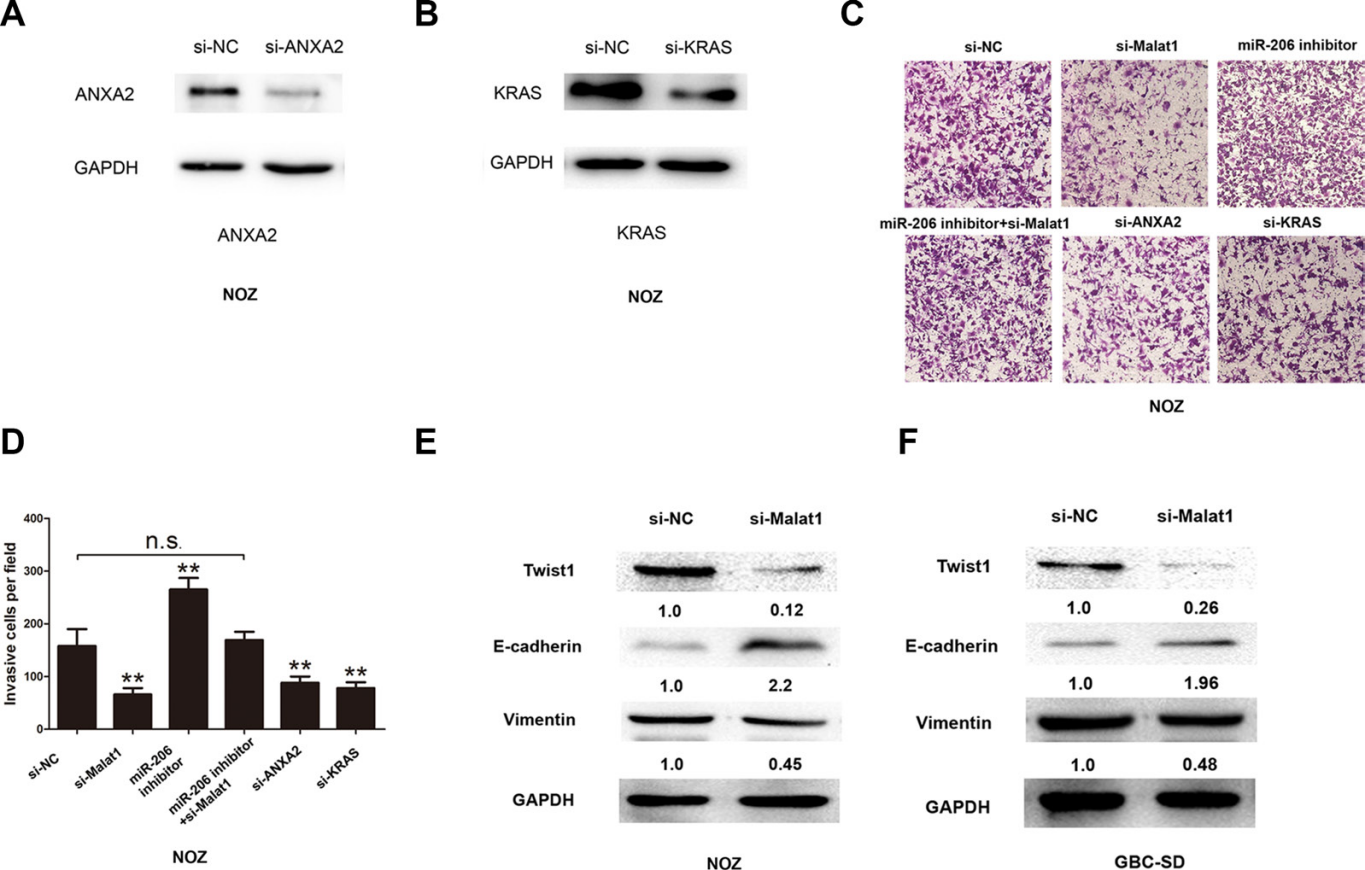
Cell invasion decreases after silencing Malat1/KRAS or ANXA2 signaling Knockdown efficacy of siRNA specific to (**A**) KRAS and (**B**) ANXA2. (**C**) Representative cell invasion images of NOZ cells transfected with si-NC, si-Malat1, miR-206 inhibitor, miR-206 inhibitor+si-Malat1, si-ANXA2 and si-KRAS, and (**D**) corresponding statistical summary. Expression of Twist, E-cadherin and vimentin after Malat1 knockdown in (**E**) NOZ cells and (**F**) GBC-SD cells. All data are represented as the mean ± S.D. from three independent experiments (**P < 0.05).

Cell invasion was investigated by trans-well assay. Knockdown of Malat1 decreased invasion of NOZ cells (Figure 6C, 6D). Cell invasion was not changed after miR- 206 inhibitor and si-Malat1 co-transfection (Figure 6C, 6D). However, cell invasion also decreased after knockdown of ANXA2 and KRAS. (Figure 6A, 6B, 6C, 6D). In addition, knocking down Malat1 in NOZ or GBC-SD cells decreased the expression of Twist and vimentin, while increasing the expression of E-cadherin (Figure 6E, 6F).

### Malat1 exhibits oncogenic activity in GBC *in vivo*

**Figure 7:**
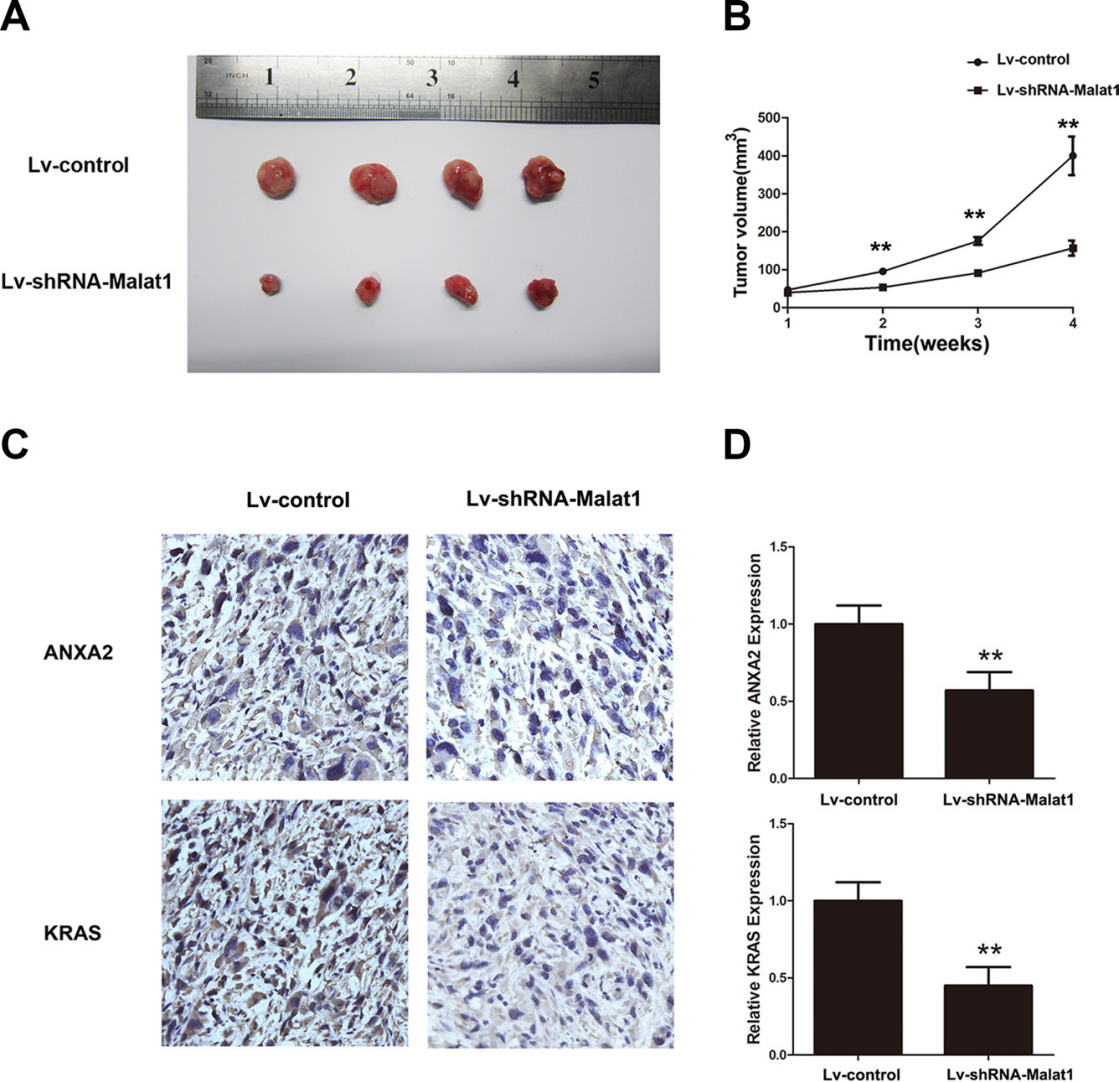
Malat1 promotes tumor growth *in vivo* (**A**) Representative image of tumor formation in nude mice four weeks after subcutaneous administration of Lv-shRNA-Malat1 and Lv-control cells. (**B**) Statistical analysis of changes in tumor volume *in vivo*. (**C**) Representative IHC pictures of ANXA2 and KRAS after Malat1 silencing *in vivo*. (**D**) Semi-quantitative analyses of IHC results for ANXA2 and KRAS after Malat1 silencing *in vivo* (**P < 0.05).

To further test Malat1′s oncogenic activity, NOZ cells stably expressing control shRNA or sh-Malat1 (Supplementary Figure S2C) were injected subcutaneously into nude mice. Tumor volumes were measured weekly and the mice were sacrificed at four weeks. Compared to the NOZ-vector group, tumor growth was reduced in the shRNA-Malat1 group (Figure 7A and 7B). Furthermore, in agreement with *in vitro* results, IHC data demonstrate that the levels of ANXA2 and KRAS are lower in the shRNA-Malat1 group than in the control group (Figure 7C and 7D). This suggests that Malat1, ANXA2 and KRAS are oncogenic in *in vivo* GBC subcutaneous tumors.

## DISCUSSION

Malat1 levels are elevated in various types of cancer [24–26]. We found that Malat1 expression is also upregulated in GBC tissues. High Malat1 levels correlate positively with tumor volume and lymphatic metastasis while correlating negatively with overall survival (OS). Knocking down Malat1 in GBC cells leads to GBC cell cycle arrest, decreased cell invasion and increased apoptosis. Our data indicate that Malat1 functions as an oncogene in GBC. However, the mechanism by which high Malat1 expression promotes tumorigenesis in gallbladder cancer is unknown.

It has been recently proposed that lncRNAs may epigenetically regulate gene expression by competing for shared miRNA response elements, thereby acting as a natural miRNA sponge that reduces the binding of endogenous miRNAs to their target genes [27]. It has been shown that Malat1 promotes aggressive Renal Cell Carcinoma by regulating EZH2 as a ceRNA for miR-205 [28]. Furthermore, Malat1 modulates Srf as a ceRNA for miR-133 in myoblast differentiation [29]. Therefore, we hypothesized that Malat1 also targets miRNAs in GBC. To test this, predictions by starbase 2.0 of miRNAs binding to Malat1 were tested by qRT-PCR. Malat1 knockdown upregulated miR-206 expression. On the other hand, overexpression of miR-206 suppressed Malat1 expression, suggesting that Malat1 is in turn negatively regulated by miR- 206. Previous studies have shown that levels of miR- 206 are abnormal in various types of cancer [30, 31, 32]. Similarly, we found that miR-206 is downregulated in GBC.

MiRNAs function through the RNA-induced silencing complex (RISC), which is comprised of many proteins [33]. Among these, Argonaut (Ago) proteins bind to mature miRNAs to promote their binding to mRNA, thereby playing a central role in RNA silencing. Here we used RIP to test whether Malat1 and miR-206 exist in the same RISC. Our results confirm that Malat1 was enriched in Ago2-containing beads, compared to controls. Moreover, dual luciferase reporter assay also confirmed that Malat1 can bind miR-206 directly. Furthermore, we performed pull-down experiments by using biotin-labeled miR-206 oligos and found that miR-206 could pull down Malat1.

Our data suggest that oncogenes KRAS and ANXA2 might be downstream targets of Malat1 and miR-206. Dual luciferase reporter assays confirmed that KRAS and ANXA2 can bind miR-206 directly at the “seed site”, which is consistent with previous reports showing that miR-206 targets KRAS and ANXA2 in pancreatic adenocarcinoma [23]. KRAS mutations are common in GBC [34], and are often linked to resistance to cetuximab, an epithelial growth factor receptor (EGFR) monoclonal antibody [35]. Here we further demonstrate that KRAS and ANXA2 levels are elevated in GBC tissues, in agreement with previous studies [36]. In addition, we find that knockdown of Malat1 inhibits KRAS and ANXA2 expression, and that this can be reversed by co-transfection of miR-206 inhibitor. These results reveal that Malat1 shares a common miRNA-responsive element with miR-206 and suggest that these molecules play a role in GBC cell proliferation, invasion and apoptosis.

In conclusion, our findings suggest that Malat1functions as a competing endogenous RNA to promote KRAS and ANXA2 expression by sponging miR-206. Therefore, Malat1 might serve as a therapeutic target as well as prognostic marker in GBC.

## MATERIALS AND METHODS

### Patients and specimens

A cohort of 30 gallbladder carcinoma tissues and relative pair-matched adjacent normal gallbladder tissues were collected postoperatively from patients who underwent liver resection at Eastern Hepatobiliary Surgery Hospital (Second Military Medical University, Shanghai, China). Consent from all patients was obtained. The study methodologies met the standard set by the Declaration of Helsinki and were approved by the Human Ethics Committee of Xinhua Hospital at Shanghai Jiao tong University (Shanghai, China). Each sample was snap-frozen in liquid nitrogen and stored at −80°C before RNA isolation. Diagnoses of GBC were given by two pathologists. All patients recruited to this study did not receive any pre-operative treatments. GBC patients were staged according to the TNM staging system (seventh edition) of the American Joint Committee on Cancer. Complete clinicopathological follow-up data for all patients were available.

### Cell culture

The non-tumorigenic human intra-hepatic biliary epithelial cell line H69 was purchased from the Health Prescience Resources Bank. GBC-SD and SGC-996 were purchased from the Cell Bank of the Chinese Academy of Science (Shanghai, China). NOZ was purchased from the Health Science Research Resources Bank (Osaka, Japan). EH-GB1 was obtained from Eastern Hepatobiliary Surgical Hospital and Institute, The Second Military University, Shanghai, China. The cell lines were cultured in Dulbecco's modified Eagle's medium (Gibco BRL, Grand Island, NY, USA), containing 10% fetal bovine serum (FBS, HyClone, Invitrogen, Camarillo, CA, USA), as well as 100 ug/ml penicillin and 100 μg/ml streptomycin (Invitrogen, Carlsbad, CA, USA). Cells were maintained at 37°C in a humidified incubator containing 5% CO2.

### RNA extraction, RT and qPCR

Total RNA (including microRNA) was extracted using TRIZOL (TaKaRa, Dalian, China), according to the manufacturers' protocol. RNA was reversed transcribed into cDNAs using the Primer-Script one step RT-PCR kit (TaKaRa, Dalian, China). The cDNA template was amplified by real-time RT-PCR using the SYBR Premix Dimmer Eraser kit (TaKaRa). Gene expression in each sample was normalized to GADPH expression. The primer sequences used were as follows: for GAPDH-forward, 5′-GTCAACGGATTTGGTCTGTATT-3′ and GAPDH-reverse, 5′-AGTCTTCTGGGTGGCAGTGAT-3′; MALAT1-forward, 5′-ATGCGAGTTGTTCTCCGTCT-3′ and MALAT1-reverse, 5′-TATCTGCGGTTTCCTCAA GC-3′. ANXA2-forward, 5′-CATGTTGGAAAGCATCAG GA-3′, ANXA2-reverse,5′-TGGAGTCATACAGCCGA TCART-3′.KRAS-forward,5′-GCAAGAGTGCCTTGAC GATA-3′, KRAS-reverse, 5′-TGACCTGCTGTGTCGA GAA-3′. TReal-time-PCR reactions were performed by the ABI7500 system (Applied Biosystems, Carlsbad, CA, USA). The relative expression fold change of mRNAs was calculated by the 2−ΔΔCt method.

### Cell transfection

SiRNAs specifically targeting MALAT1 were synthesized by Shanghai Gene Pharma Co, Ltd. SiRNA sequences for MALAT1: sense-1, 5′-CACAGGGAAAG CGAGTGGTTGGTAA-3′; antisense-1, 5′-TTACCAAC CACTCGCTTTCCCTGTG-3′; sense-2, 5′-GAGGUGUA AAGGGAUUUAUTT-3′; antisense-2, 5′-AUAAAUCCC UUUACACCUCTT-3′; sense-3, 5′-GGCCAAAUGUUG AAGUUAATT-3′; antisense-3, 5′-UUAACUUCAACAU UUGGCCTT-3′. Negative siRNA sense: 5′-GGCCUAAAG UAGUAGCUAUTT-3′; antisense, 5′-AUAGCUACUACU UUAGGCCTT-3′. Sequences for ANXA2 siRNA, 5′-GAACUUGCAUCAGCACUGATT-3′; Sequences for KRAS siRNA, 5′-CUAUGGUCCUAGUAGGAAATT-3′. The concentration of relative siRNA, mimic and inhibitor were 20 μM, while the working concentration was 20 nM. SiRNA Plasmids were transfected into cells using Lipofectamine TM 2000 (Invitrogen, Carlsbad, CA, USA) and were incubated for 48 h according to the manufacturer's instructions. The working concentration of relative plasimids was 100 nM. MiR-206 mimics and inhibitor were transfected into NOZ cells using Lipofectamine TM 2000 (Invitrogen, Carlsbad, CA, USA). The following short hairpin RNA (shRNA) was used to target human MALAT1: sense: 5′-CACAGGGAAA GCGAGTGGTT GGTAA-3′; antisense: 5′-TTACCAACCA CTCGCTTTCC CTGTG-3′. The sequence of the negative control shRNA was 5′-TTCTCCGAAC GTGTCACGT-3′ The shRNAs were synthesized and inserted into the pHBLV-U6 lentivirus core vector containing a ZS green fluorescent protein (Hanbio, Shanghai, China).

### Trans-well invasion assay

Trans-well invasion assay was performed using Matrigel-coated (BD, Franklin Lakes, NJ, USA) filters in 24- well plates. Cells were trypsinized and seeded onto the upper chambers of the trans-well (1 × 10^5^ cells/well) in serum-free DMEM medium. The lower chambers of the trans-well were filled with DMEM medium (including 10% fetal bovine serum). The chambers were incubated at 37°C and 5% CO2 for 24 h. At the end of incubation, cells on the upper surface of the filter were removed by wiping with a cotton swab. Cells migrating through the filter to the lower surface were fixed with 4% paraformaldehyde for 10 min and stained with 0.1% crystal violet for 10 min. Cells washed 3 times in PBS were visualized using a phase-contrast microscope (Olympus, Tokyo, Japan) and counted from randomly chosen fields.

### Western blot analysis

Cells were lysed in RIPA buffer containing fresh protease and phosphatase inhibitor cocktails (Sigma) by incubating for 20 min at 4°C. Protein concentration was determined using the BCA assay (Beyotime Biotechnology, China) according to manufacturer's instructions. Samples were then subjected to SDS-PAGE electrophoresis and transferred to polyvinylidenedifluoride (PVDF) membranes (Millipore, Billerica, MA) and incubated on a shaker overnight at 4°C with primary antibodies against KRAS (1:500, Proteintech, USA), ANXA2 (1:1000, Abcam, USA), GAPDH (1:1000, Cell Signaling Technology), Twist1 (1:1000, Abcam, USA), E-cadherin (1:500, Santa Cruz, USA) and Vimentin (1:500, Santa Cruz, USA). Horseradish peroxidase (HRP)-conjugated secondary antibody (1:1000, Abcam) was incubated at room temperature for 1.5 h. Blots were developed using enhanced chemiluminescence detection reagents and scanned with a Molecular Imager system (Bio-Rad).

### Dual-luciferase reporter assay

Human HEK293T cells (2.0 assayd with a Molecular Imager were co-transfected with 150 ng of either empty, pmirGLO-NC, pmirGLO-MALAT1-wt or pmirGLO-MALAT1-mut (Sangon biotech, China). Two ng of pRL-TK (Promega, Madison, WI, USA) were also co-transfected with miR-206 mimics or miRNA NC into HEK293T cells by using Lipofectamie 2000 (Invitrogen, USA). PmirGLO-KRAS-wt and pmirGLO-ANXA2-wt (Sangon biotech, China) were transfected into HEK293T cell by Lipofectamine-mediated gene transfer. The relative luciferase activity was normalized to Renilla luciferase activity 48 h after transfection. Transfection was repeated in triplicate.

### RNA immunoprecipitation (RIP)

RIP assay was performed using the EZ-Magna RIP™ RNA-Binding Protein Immunoprecipitation Kit (Millipore, MA, USA) according to the manufacturer's instructions. NOZ cells at 80–90% confluency were scraped off and lysed in complete RIP lysis buffer. Then, 100 μl of whole cell extract were incubated with RIP buffer containing magnetic beads conjugated with human anti-Ago2 antibody (Cell Signaling, USA), negative control normal mouse IgG (Millipore) and positive control SNRNP70 (Millipore). The co-precipitated RNAs were detected by reverse transcription PCR. Total RNAs (input controls) and IgG were assayed simultaneously to test whether the detected signals resulted from RNAs specifically binding to Ago2.

### Pulldown assay with biotinylated miRNA

NOZ cells were transfected with Biotinylated miR-206 or biotinylated mutant miR-206 or biotinylated NC (synthesized by Shanghai Gene Pharma Co, Ltd) using Lipofectamine 2000 according to the manufacturer's instructions. The final concentration of each biotinylated miRNA was 20 nM. The cell lysates were collected 48 h after transfection and incubated with M-280 streptaviden magnetic beads (Invitrogen, San Diego, CA, USA) as described previously [37]. The bound RNAs were purified using TRIzol reagent (TAKALA) for further RT–qPCR analysis.

### Flow cytometric analysis

Negative control cells or cells transfected with the desired plasmid were plated in six-well plates. After 48 h incubation, the cultures were incubated with propidium iodide for 30 min in the dark. The percentage of cells in G0/G1, S and G2/M phases of the cell cycle was measured using a flow cytometer (FACS Calibur, BD Biosciences, San Jose, CA, USA) after propidium iodide staining. Cultures were also analyzed for cell apoptosis after double staining with FITC-Annexin V and Propidium iodide (PI). The cells were analyzed with a flow cytometer (FACScan^®^; BD Biosciences) equipped with CellQuest software (BD Biosciences).

### Immunohistochemistry (IHC)

Tumor specimens from nude mice were fixed in 4% paraformaldehyde and then embedded in paraffin. Sections were used for KRAS (1:50, Proteintech, USA) and ANXA2 (1:100, Abcam, USA) analyses. The samples were incubated at 4°C overnight with primary antibodies against ANXA2 and KRAS. The sections were treated with secondary antibody for 30 min at room temperature and stained with diaminobenzidine (DAB) until brown granules appeared. Sections were blindly evaluated by two pathologists using light microscopy.

### Xenograft mouse model

NOZ cells (1 × 10^6^) stably expressing control shRNA or shRNA-MALAT1 were subcutaneously injected into either side of flank area of 4-week-old male athymic nude mice (*n* = 4 mice per group). Tumor volumes were measured (0.5 × length × width^2^) in mice on a weekly basis. After 4 weeks, the nude mice were sacrificed and the tumor tissues were excised and fixed in 4% paraformaldehyde solution for further study. All animal experiments were performed in the animal laboratory center of Xinhua Hospital and in accordance with the Guide for the Care and Use of Laboratory Animals published by the US National Institutes of Health (NIH publication number 85–23, revised 1996). The protocol was approved by the Animal Care and Use committee of Xinhua Hospital.

### Statistical analysis

Data are presented as mean ± standard deviation (S.D.). *T*-tests were used to measure the statistical significance of differences. Pearson's correlation coefficient was used to analyze the correlation between Malat1 and miR-206 in cancer tissues. Survival plots were generated by Kaplan-Meier analysis, and the log-rank test was used to assess the significance of the differences. *P* < 0.05 was considered to be significant.

## SUPPLEMENTARY MATERIALS FIGURES AND TABLES


