# Supplementary material for: Long non-coding RNA Malat1 promotes gallbladder cancer development by acting as a molecular sponge to regulate miR-206
Source: Oncotarget. 2016 May 13;7(25):37857–67. doi: 10.18632/oncotarget.9347 (PMC5122355; doi:10.18632/oncotarget.9347)
Supplement: Supplementary file 1 [file oncotarget-07-37857-s001.pdf]

Long non-coding RNA Malat1 promotes gallbladder cancer development by acting as a molecular sponge to regulate miR-206

Supplementary Materials

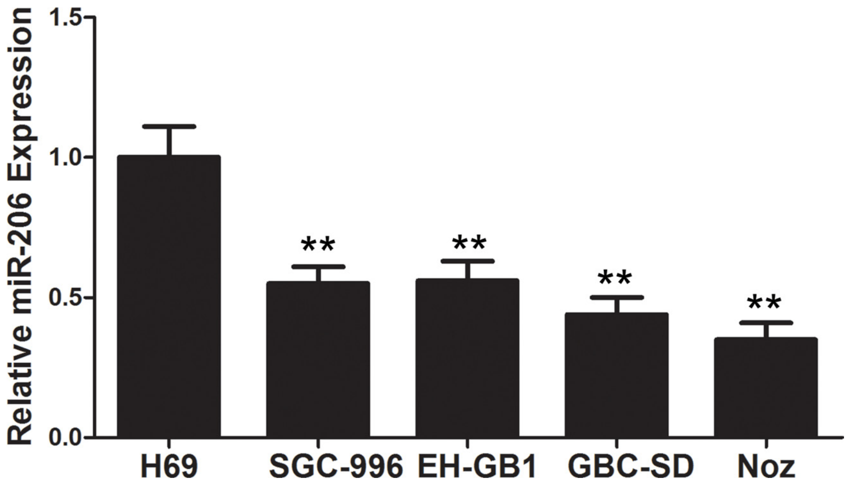

Supplementary Figure S1: MiR-206 expression in GBC and normal biliary epithelial cell lines: Relative expression levels of miR-206 in normal cell H69 or four indicated GBC cells, $**P < 0.05$.

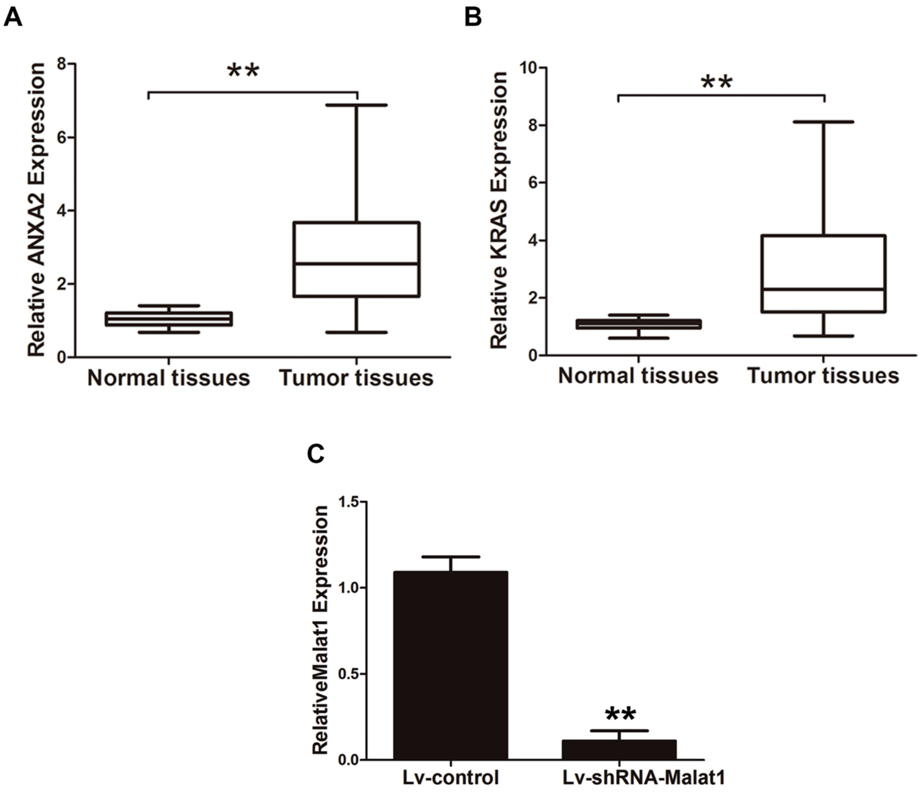

Supplementary Figure S2: (**A**) The transcriptional levels of ANXA2 in GBC tissue compared to normal match gallbladder tissue, $**P < 0.05$. (**B**) The transcriptional levels of KRAS in GBC tissue compared to normal match gallbladder tissue, $**P < 0.05$. (**C**) The expression levels of Malat1 transfected with Lv-shRNA-Malat1 compared to the Lv-control group in NOZ cells. $**P < 0.05$.

Supplementary Table S1: MiRNA fold change in responses to Malat1 knockdown in NOZ cells

miRNA	fold change
hsa-miR-370-3p	0.98
hsa-miR-503-5p	1.25
hsa-miR-378a-3p	1.41
hsa-miR-506-3p	1.22
hsa-miR-135b-5p	1.55
hsa-miR-203a	1.38
hsa-miR-216a-5p	1.14
hsa-miR-26b-5p	0.88
hsa-miR-185-5p	1.21
hsa-miR-206	2.78
hsa-miR-455-5p	1.1
hsa-miR-363-3p	2.4
hsa-miR-146b-5p	0.91
hsa-miR-378c	0.99
hsa-miR-141-3p	1.65
hsa-miR-26a-5p	1.33
hsa-miR-135a-5p	1.52
hsa-miR-494-3p	1.35
hsa-miR-194-5p	1.88
hsa-miR-140-5p	1.44
hsa-miR-22-3p	1.2
hsa-miR-142-3p	0.9
hsa-miR-143-3p	1.78
hsa-miR-145-5p	1.12
hsa-miR-202-3p	1.89
hsa-miR-491-5p	0.78
hsa-miR-485-5p	1.66
hsa-miR-154-5p	1.46
hsa-miR-211-5p	1.53
hsa-miR-101-3p	1.34
hsa-miR-613	1.58
hsa-miR-519d-3p	1.48
hsa-miR-204-5p	1.25
